# Mesenchymal stem cells show functional defect and decreased anti-cancer effect after exposure to chemotherapeutic drugs

**DOI:** 10.1186/s12929-018-0407-7

**Published:** 2018-01-19

**Authors:** Chinnapaka Somaiah, Atul Kumar, Renu Sharma, Amit Sharma, Trishna Anand, Jina Bhattacharyya, Damodar Das, Sewali Deka Talukdar, Bithiah Grace Jaganathan

**Affiliations:** 1 Stem Cell and Cancer Biology Group, Department of Biosciences and Bioengineering, Indian Institute of Technology Guwahati, Guwahati, India; 2 Department of Hematology, Gauhati Medical College and Hospital, Guwahati, India

**Keywords:** Bone marrow stroma, Chemotherapy, Chemoprotection, Leukemia, Osteoblasts, IL6, FGF2

## Abstract

**Background:**

Mesenchymal stem cells (MSC) are used for several therapeutic applications to improve the functions of bone, cardiac, nervous tissue as well as to facilitate the repopulation of hematopoietic stem cells. MSC give rise to the non-hematopoietic stromal cells of the bone marrow and are important for the maintenance of normal hematopoiesis. Chemotherapeutic drugs used for treatment of leukemia extensively damage the stromal cells and alter their gene expression profiles.

**Methods:**

We determined the changes in adipogenic, osteogenic differentiation, phenotypic and gene expression in MSC during treatment with chemotherapeutic drugs cytarabine, daunorubicin and vincristine. We also tested anti-cancer effects of drug treated MSC on leukemia cells.

**Results:**

Treatment with the chemotherapeutic drugs resulted in functional defects in MSC, leading to reduced proliferation, osteogenic and adipogenic differentiation. The drug treated MSC also showed decreased expression of cell surface receptors, and the changes in proliferation, phenotype and differentiation defect was partially reversible after withdrawing the drugs from the cells. The drug treated MSC showed increased expression of cytokines, IL6, FGF2 and TNFA but reduced levels of differentiation markers SOX9 and ACTC1. Drug treated MSC also contributed to reduced anti-cancer effects in leukemia cells.

**Conclusions:**

Chemotherapeutic drug treatment altered the phenotype, osteogenic and adipogenic differentiation potential of MSC and modified the gene expression profile of the cells to render them more chemoprotective of the leukemic cells. Thus, additional therapeutic efforts to target the stromal cell population will help in preventing chemoresistance, disease relapse in leukemia and to maintain a healthy bone marrow stroma.

**Electronic supplementary material:**

The online version of this article (10.1186/s12929-018-0407-7) contains supplementary material, which is available to authorized users.

## Background

The bone marrow derived MSC have the ability to differentiate into several cell types and have gained importance in regenerative medicine, tissue engineering and immune modulation [17, 24, 25]. MSC contribute to the development of non-hematopoietic stromal cells in the bone marrow. The stromal cells present in the bone marrow are important for maintenance of normal and malignant hematopoietic cells [31]. Chemotherapeutic drugs used for leukemia therapy not only target the cancer cells, but also affect the cells of the hematopoietic microenvironment. Chemotherapeutic treatment for hematologic malignancies as well as other cancers have been shown to damage the bone marrow microenvironment cells and MSC in vitro and in vivo [7, 11, 14, 20–23].

When allogeneic MSC were utilized for co-injection with bone marrow cells to improve the engraftment percentage, the allogeneic MSC did not engraft long-term, however the recipient MSC supported the hematopoietic cells [1, 9]. Thus, defective stromal cells in the bone marrow might affect the long-term recovery of hematopoiesis after chemotherapy or allogeneic hematopoietic stem cell transplantation where the recipient stromal cells have to support the hematopoietic stem cells and recovery of hematopoiesis. When MSC are required for autologous transplantation to mediate tissue repair or regeneration, it is important to understand the changes sustained by the MSC due to exposure to the chemotherapeutic drugs.

Another important aspect that must be understood is, whether the pre-exposure of MSC to chemotherapeutic drugs affect their ability to support the leukemic cells during chemotherapy since MSC have been shown to have functional aberrations and protect leukemia cells during chemotherapy [12, 13]. Chemotherapy treatment for leukemia is performed in several cycles and any change in the gene expression profile of the stromal cells which render them more supportive of the leukemic cells will result in an unfavorable outcome. Thus, understanding the effect of chemotherapeutic drugs on the functional and gene expression properties of MSC will help in improving the treatment strategies for leukemia and to avoid relapse of the disease.

In the current study, we determined the effect of chemotherapeutic drugs cytarabine (CYT), daunorubicin (DAU) and vincristine (VIN) which are frequently utilized for leukemia treatment, on the properties of MSC. The cell surface marker expression, differentiation potential and gene expression profiles were determined for the chemodrug treated MSC. We found that the chemotherapeutic drugs significantly reduced the adipogenic and osteogenic differentiation ability of MSC, however the cells partially recovered their differentiation potential after the removal of the drug. The drug pre-treated MSC were more supportive of the leukemic cells during the drug treatment which was accompanied by increased expression of IL6, IL8, FGF2 or TNFA.

## Methods

### Chemicals and reagents

Dulbecco’s modified eagle’s medium (DMEM), Oil red O, alizarin red, dexamethasone, iso butyl methyl xanthine, indomethacin, insulin, β- glycerophosphate, ascorbic acid and basic fibroblast growth factor were purchased from Sigma Aldrich (Steinheim, Germany). Tissue culture plastic plates and flasks were from Eppendorf (Germany). Fluorescent conjugated anti-human antibodies were from BD biosciences (Germany). Fetal bovine serum (FBS) and real-time PCR reagents were purchased from Thermo Fisher scientific (USA).

### Bone marrow MSC

MSC were isolated from the bone marrow of patients who have been referred to the Hematology Department of Gauhati medical college hospital for diagnosis. The samples were obtained after informed consent as per hospital ethical committee guidelines. The bone marrow cells were subjected to RBC lysis with ammonium chloride solution (0.15 M, pH 7.3) on ice and the resulting cells were seeded in tissue culture plates for isolation of MSC as described earlier [28].

### Drug treatment of MSC

MSC were seeded in tissue culture plates and treated with the chemotherapeutic drugs cytarabine (10μM), daunorubicin (0.1μM) or vincristine (0.1μM) for 48 h unless otherwise mentioned. The media containing the drug was removed and live cell percentage was determined by trypan blue exclusion counting. The cells were seeded in fresh plates with complete media without the drugs and allowed to proliferate. The recovered cells were utilized for further experiments.

### Adipogenic and osteogenic differentiation

Adipogenic and osteogenic differentiation of MSC was performed as described earlier [18, 27]. For adipogenic differentiation, the cells were cultured in DMEM media with 10% serum supplemented with dexamethasone, isobutylmethylxanthine, indomethacin and insulin. For osteogenic differentiation, media containing 10% serum and dexamethasone, β-glycerophosphate, and ascorbic acid were added to the cells. The cells were differentiated for 21 days in the induction media and osteogenic differentiation was determined by staining for alizarin red and adipogenic differentiation by oil red-O staining. The alizarin red levels were quantified by eluting with 10% (*w*/*v*) cetylpyridinium chloride in 10 mM sodium phosphate, and absorbance measurement at 562 nm and Oil red O stain was extracted from the cells with 100% isopropanol and quantified by absorbance measurement at 500 nm.

### Phenotyping

The cell surface protein expression was analyzed by flow cytometry. MSC were trypsinized and stained with fluorescent dye conjugated anti-human antibodies for 30 min at 4°C. The cells were washed, stained with propidium iodide (PI) for live/dead discrimination and analyzed with FACS caliber (BD Biosciences).

### Co-culture of leukemia cells with stromal cells

HL60 or THP1 leukemic cell line (1x10^5^cells/ml) was added to drug pre-treated or untreated MSC seeded in tissue culture plates. The cells were cultured for 48 h and indicated chemotherapeutic drugs were added to the co-culture at the indicated concentrations. After 48 h of treatment, the leukemic cells in suspension were collected from the co-culture without disturbing the MSC layer. Adherent leukemia cells were collected by brief trypsinization (30s) while monitoring the detachment of leukemic cells from the stromal cells under microscope. Both the suspension and adherent leukemia cells were further processed for apoptosis analysis.

### Apoptosis analysis

Apoptosis analysis was performed by staining the cells with Annexin-V and PI (Thermo Fisher Scientific) according to the manufacturer’s instructions. The cells were washed with ice-cold PBS, stained with anti -annexin V antibody and propidium iodide and incubated in dark for 15 mins at room temperature. The stained cells were analyzed with FACS Calibur.

### Gene expression analysis

Gene expression analysis was performed by real-time PCR. Total RNA was extracted using TriZol reagent (Thermo Fisher Scientific). RNA was reverse transcribed using superscript III reverse transcriptase and Oligo dT primers. The GAPDH, PPARG and OCN primer sequences were as described earlier [28]. Other primer sequences are given in supplementary information (Additional file 1: Table S1). Real-time PCR was performed with Power SyBr Green reagents in an ABI 7500 real-time PCR machine (Thermo Fisher Scientific). The gene expression levels in each sample was normalized to their respective GAPDH expression level. The fold change in the expression levels compared to the control was calculated using ΔΔCt method.

### Data analysis

Flow cytometric data was analyzed using FlowJo software (FlowJo, LLC). Geometric mean fluorescence intensity (MFI) was calculated to detect the changes in the expression levels. Statistical analysis was performed using SPSS software and student T test was used to compare the difference between treated and untreated samples. Variation between different MSC samples in their gene expression profiles was determined by Mann-Whitney non-parametric variables test.

## Results

### Chemotherapeutic drug treatment affects the morphology and proliferation of MSC

**Fig. 1:**
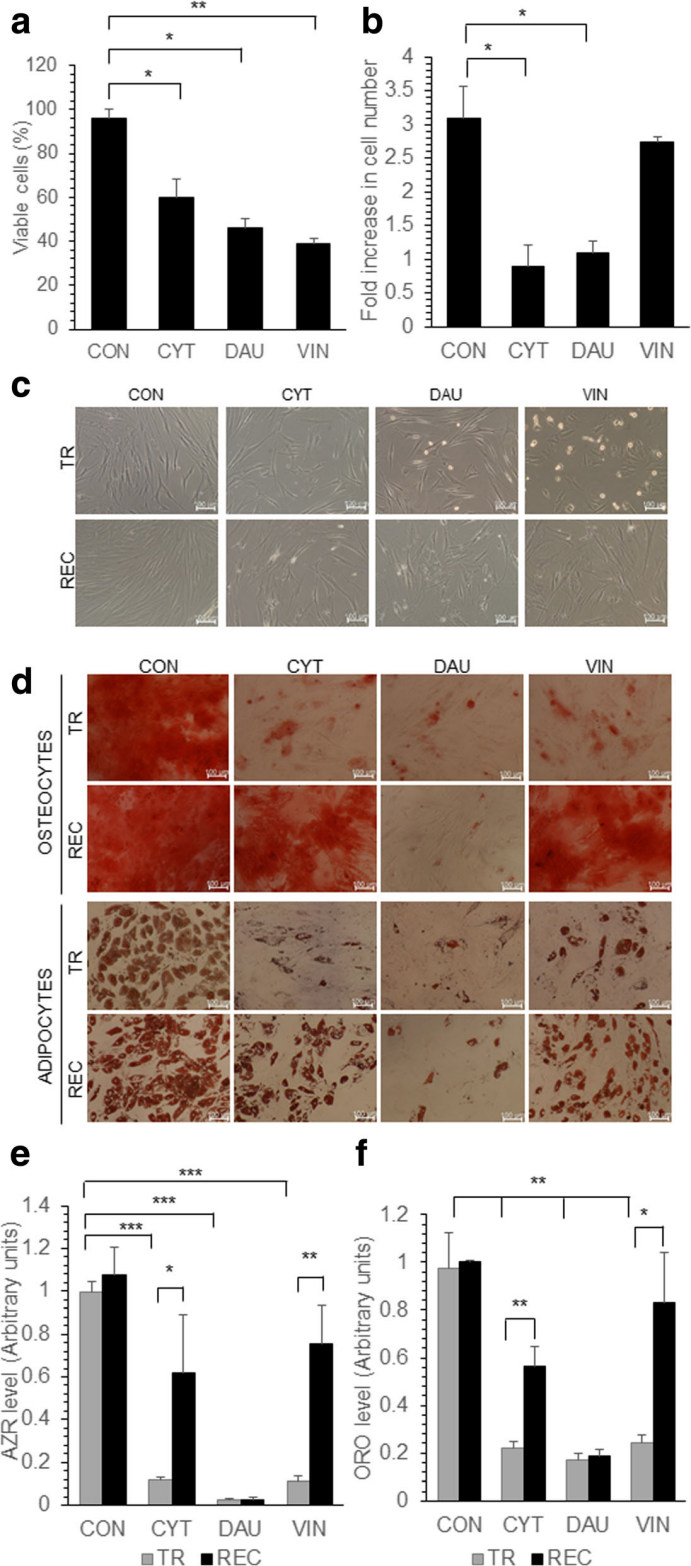
Effect of chemotherapeutic drugs on MSC proliferation and differentiation. MSC were either untreated (CON) or treated with CYT (10μm), DAU (0.1μm) and VIN (0.1μm) for 48 h. **a** The viable cells were counted microscopically using trypan blue exclusion and the percentage of viable cells was calculated. **b** Equal number of drug treated MSC were seeded in complete media without the drug, the viable cell number was counted after 7 days and the fold increase was calculated. **c** Representative microscopic images showing the morphology of MSC treated with the drugs for 48 h (TR) and drug treated cells allowed to proliferate in the drug free media for 7 days after drug treatment (REC). **d** Representative microscopic images showing the differentiation of MSC into osteoblasts and adipocytes either immediately after treatment with drugs (TR) or after the recovery of cells in drug free media for 7 days (REC). Osteogenic or adipogenic differentiation media was added to the cells to induce them to differentiate into respective lineage. **e**, **f** Alizarin red (AZR) or oil red O (ORO) quantification of MSC differentiated immediately after drug treatment (TR) or after recovery in drug free media (REC). AZR and ORO staining was done to detect osteogenic and adipogenic differentiation respectively. Values are mean ± SD, *n* = 3 samples. *p < 0.05, **p < 0.005, ***p < 0.0005

In order to understand the effect of chemotherapeutic drugs used for leukemia treatment on the bone marrow MSC, the cells were treated with the chemotherapeutic agents CYT, DAU and VIN. When MSC were treated with CYT, DAU or VIN, there was a significant decrease in the cell proliferation (Fig. 1a). VIN treated cells had the lowest viable cell number compared to the CYT or DAU treated cells (38.8 ± 2.4% in VIN treated cells, 59.7 ± 8.7% in CYT treated cells, 45.8 ± 4.2% in DAU treated cells versus 95.8 ± 4.1% in control). VIN treatment affected the cell morphology significantly where the spindle shaped cells assumed a flattened morphology. Reduced proliferation was observed even 7 days after the drug removal in CYT and DAU treated MSC whereas VIN treated MSC recovered their proliferation capacity and proliferated at similar rates as that of the control cells (Fig. 1b). Thus, the proliferation inhibition of VIN was reversible in MSC and effect of CYT and DAU were not completely reversible although the cells partially regained their proliferation capacity. Moreover, after culturing the cells in the drug free media, the cells treated with all the drugs CYT, DAU and VIN assumed normal spindle shape (Fig. 1c).

### Changes in osteogenic, adipogenic differentiation and phenotype was partially reversible after chemotherapeutic drug treatment

MSC give rise to osteoblasts and adipocytes when induced with specific factors. MSC derived osteoblasts maintain the hematopoietic stem cells in the bone marrow [31], therefore, efficient osteoblastic differentiation of MSC treated with chemotherapeutic drug is important for tissue engineering applications as well as for the recovery of the normal hematopoiesis in the patient during remission. MSC treated with CYT, DAU and VIN were induced to differentiate into osteoblasts and adipocytes immediately after drug treatment or after culturing the cells in the drug free media for 7 days. Both osteogenic and adipogenic differentiation was significantly reduced in drug treated MSC, where there was an average ten-fold reduction in osteogenic differentiation and five-fold reduction in adipogenic differentiation potential of drug treated MSC (Fig. 1d-f). When MSC were allowed to proliferate in the drug free media post treatment, they showed higher osteogenic and adipogenic differentiation ability compared to MSC induced to differentiate soon after drug treatment (Fig. 1e, f). Reduction in osteogenic and adipogenic differentiation was completely reversed in VIN treated MSC when they were cultured in VIN free media prior to induction of differentiation. CYT treated MSC partially regained their osteogenic and adipogenic differentiation potential whereas inhibition of osteogenic and adipogenic differentiation due to DAU treatment was not fully reversible (Fig. 1d-f).

**Fig. 2:**
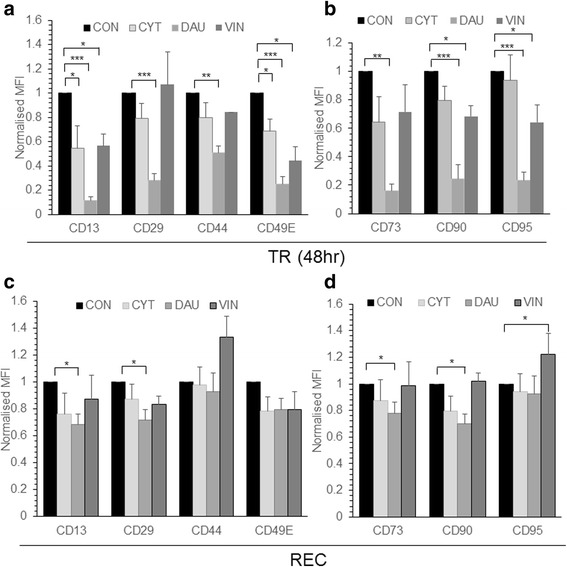
Phenotype of drug treated MSC. MSC were treated with CYT (10μM), DAU (0.1μM) and VIN (0.1μM) for 48 h. **a**, **b** Expression of indicated cell surface markers after CYT, DAU, VIN treatment or in control (CON) untreated cells was analyzed by flow cytometry (TR). **c**, **d** Drug treated and control MSC were allowed to recover in drug free media for 7 days (REC) and the cell surface marker expression was analyzed. MFI represents mean fluorescent intensity of the markers analyzed and it is normalized to their respective isotype controls. Values are mean ± SE, *n* = 3–5 samples. *p < 0.05, **p < 0.005, ***p < 0.0005

Further MSC treated with CYT, DAU and VIN were analyzed for changes in cell surface expression of integrins CD13, CD29, CD49E, cell surface markers CD44, CD73, CD90 and death receptor CD95. There was a significant decrease in the expression of all cell surface markers after DAU treatment whereas CYT and VIN treatment significantly reduced the expression of CD13 and CD49E (Fig. 2a). VIN treatment also resulted in significant reduction of CD90 and CD95 cell surface expression (Fig. 2b). When MSC were cultured in the drug free media post-drug treatment for 7 days, CYT and VIN treated MSC fully recovered their cell surface marker expression except there was a significantly high expression of CD95 in VIN treated MSC (Fig. 2c, d). On the other hand, although DAU treated MSC recovered the cell surface expression levels of CD44, CD49E and CD95, it showed a significantly less cell surface expression of CD13, CD29, CD73 and CD90 after recovery (Fig. 2c, d) compared to control MSC. Nevertheless the expression levels of CD13 (MFI: 0.12 in treated vs 0.68 in recovered; *p* = 0.00209), CD29 (MFI: 0.28 in treated vs 0.72 in recovered; *p* = 0.0029), CD73 (MFI: 0.16 in treated vs 0.78 in recovered; *p* = 0.00053) and CD90 (MFI: 0.24 in treated vs 0.698 in recovered; *p* = 0.0119) in MSC cultured in drug free media post drug treatment were significantly higher than those observed in MSC immediately after the DAU treatment (Fig. 2).

### Drug treated MSC provided higher chemoprotection for leukemia cells

**Fig. 3:**
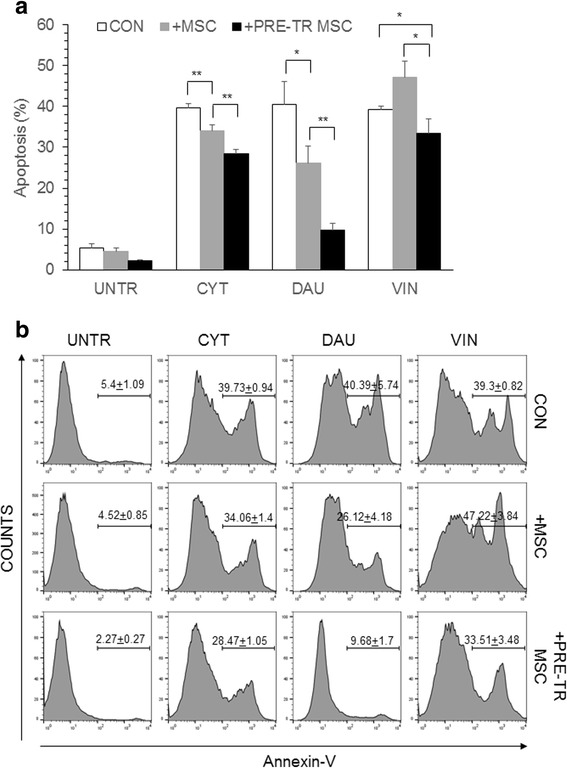
Chemoprotection of leukemia cells by drug treated MSC. **a** HL60 leukemia cells were cultured for 48 h in the absence of MSC (CON) or, in the presence of MSC (+MSC) or in the presence of drug pre-treated MSC (+PRE-TR MSC). The cells were treated with CYT (10μM), DAU (0.1μM) and VIN (0.1μM) for 48 h and apoptosis percentage was analyzed flow cytometrically by staining with annexin-V and PI. Values are mean ± SD, *n* = 3 samples. **b** Representative flow cytometric plots showing the apoptosis percentage in drug-treated HL60 as shown in (**a**). *p < 0.05, **p < 0.005

In order to understand whether treatment with chemotherapeutic drugs render the MSC more supportive of the leukemia cells, apoptosis of leukemia cells were analyzed during co-culture with MSC. Leukemia cell lines HL60 was co-cultured with MSC pre-exposed to chemotherapeutic drugs CYT, DAU or, VIN or in the presence of untreated MSC. CYT, DAU or VIN was added to the cells in co-culture and apoptosis percentage of HL60 was analyzed. HL60 in co-culture with MSC were treated with the same drug to which MSC were pre-exposed, for instance, HL60 co-cultured with MSC were treated with CYT if the MSC were pre-exposed to CYT and so on. CYT, DAU and VIN treatment significantly induced apoptosis in the control HL60 cells (Fig. 3). As reported by others [5], co-culture with MSC significantly reduced the chemosensitivity of HL60 to the chemotherapeutic drugs except during VIN treatment (Fig. 3). However, co-culture of HL60 or THP1 leukemic cells with drug pre-treated MSC significantly reduced their apoptosis percentage during chemotherapeutic drug treatment suggesting a higher chemoprotective effect of drug treated MSC (Fig. 3, Additional file 2: Figure S1). Thus, pre-exposure of MSC to chemotherapeutic drugs render them more supportive of the leukemia cells during chemotherapeutic treatment.

**Fig. 4:**
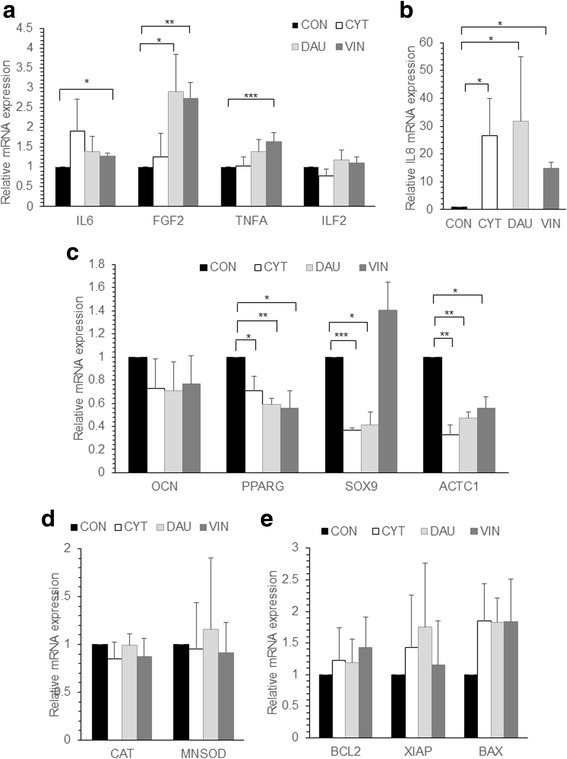
Gene expression of chemo drug treated MSC. **a**-**e** MSC were left untreated (CON) or treated with CYT, DAU or VIN for 48 h. After treatment, the cells were cultured in drug free media for 7 days and total RNA was extracted from the cells and reverse transcribed into cDNA. Semi-quantitative real-time PCR was done to analyze the mRNA expression levels of the indicated genes and the values were normalized to GAPDH expression levels in the respective samples. Values are mean ± SE, n = 3–4 samples. *p < 0.05, **p < 0.005, ***p < 0.0005

**Fig. 5:**
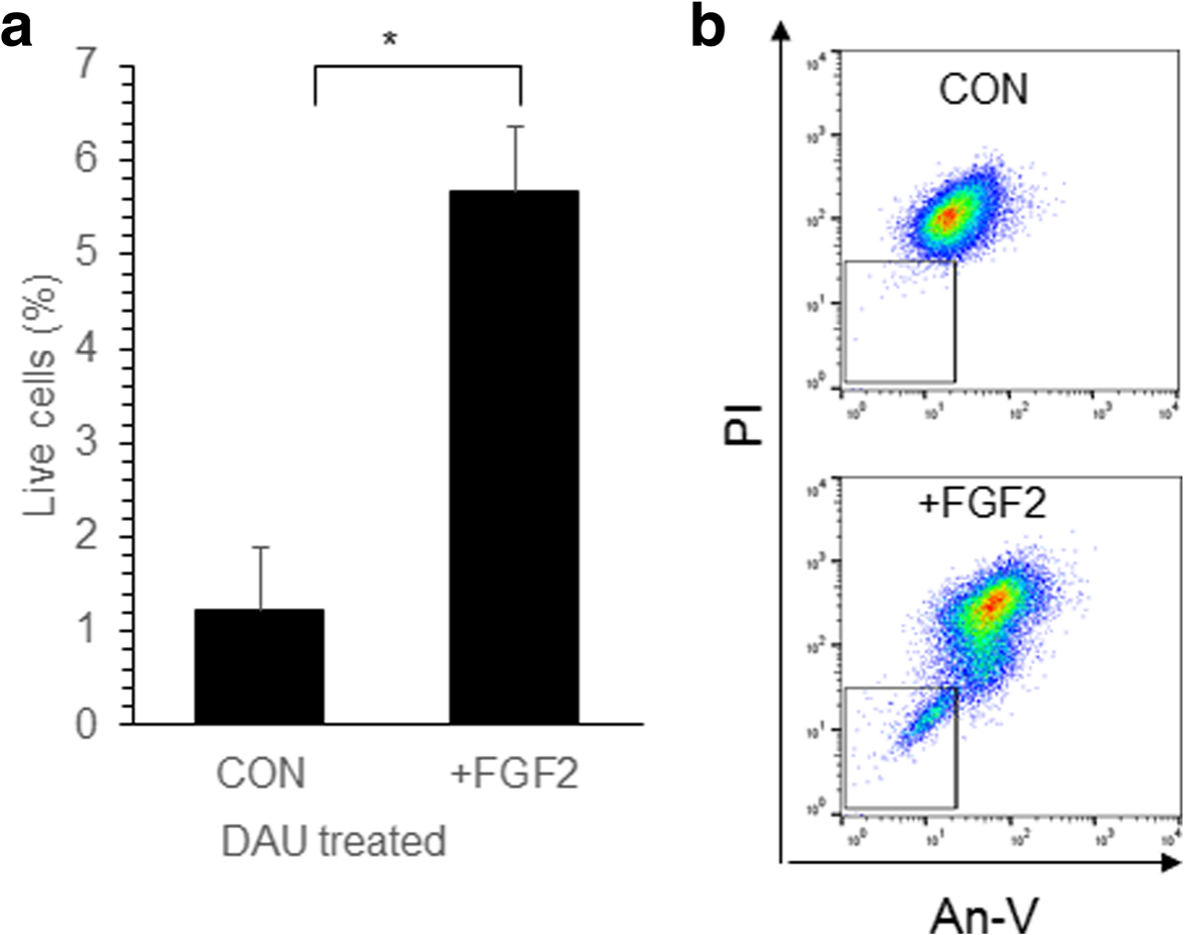
FGF2 increased the survival of leukemia cells during chemotherapeutic drug treatment. **a** THP1 leukemia cells were treated with DAU (50 nM) for 4 days in the presence (+FGF2) or absence (CON) of FGF2 (10 ng/ml). Percentage of live and dead cells was analyzed by annexin-V and PI staining. Values are mean ± SE, n = 3 samples. **b** Representative flow cytometric plots showing the live cell percentage for the plots shown in (**a**). *p < 0.05

To understand the chemoprotective effect of drug treated MSC further, the gene expression changes in CYT, DAU and VIN treated MSC were analyzed. Since the MSC after drug treatment were highly apoptotic (Fig. 1a), the gene expression changes was analyzed in MSC that were allowed to recover for 7 days after drug removal. DAU treated MSC showed significantly high expression of FGF2; VIN treated MSC had significantly high IL6, FGF2 and TNFA mRNA expression levels (Fig. 4a). A significantly high IL8 expression was observed in MSC pre-treated with all the different drugs whereas no change in ILF2 levels were observed (Fig. 4b). The expression of osteogenic specific gene OCN was unaffected in all conditions whereas the adipogenic differentiation gene PPARG was downregulated in drug treated cells (Fig. 4c). Chondrogenic differentiation gene SOX9 was significantly reduced in CYT and DAU treated MSC whereas it was unaffected in VIN treated cells (Fig. 4c). Cardiac actin gene (ACTC1) was significantly downregulated in MSC treated with all the different drugs (Fig. 4c). No significant difference in expression was seen in oxidative stress response genes MNSOD, CAT (Fig. 4d) or apoptosis related genes BCL2, XIAP or BAX (Fig. 4e) was seen. To check whether the increased expression of FGF2 observed in drug treated MSC might contribute to the chemoprotection of leukemia cells, THP1 leukemia cell line was cultured and treated with DAU in the presence of FGF2. We found a significant increase in the percentage of live cells when FGF2 was present during DAU treatment compared to the control (Fig. 5). Thus, chemotherapeutic drugs modify the MSC gene expression profile which in turn might chemoprotect leukemia cells.

## Discussion

A healthy bone marrow microenvironment is necessary for repopulation of normal hematopoietic cells after chemotherapeutic treatment. Several studies have shown that co-injection of MSC with the hematopoietic cells significantly increased the repopulation and reconstitution of the recipient hematopoietic system [1, 9]. However, the donor MSC failed to engraft long-term in the recipient and so the hematopoietic recovery and functioning were carried out by the host MSC [1, 9]. Thus, it is important to understand how chemotherapy affects the MSC characteristics, especially the osteogenic differentiation ability and expression of pro-survival cytokines which is critical to maintain the hematopoietic stem cells. Our study has shown that each chemotherapeutic agent had varied effect on the MSC phenotype, differentiation ability and gene expression. Although a combination of drugs are used during chemotherapeutic treatment for leukemia, understanding the effect of individual drug at physiological concentration is important. All the chemotherapeutic drugs induced apoptosis of MSC and decreased proliferation was observed even after the removal of the chemotherapeutic drugs. Similar anti-proliferative and apoptotic effects were reported in MSC after treatment with doxorubicin [22] and bleomycin [20]. However, adipose derived MSC were reported to be resistant to toxic effects of cisplatin, vincristine and camptothecin [3, 15] and bone marrow derived MSC were resistant to cisplatin [19] and paclitaxel [4] induced apoptosis. In our study, we observed a significant reduction in osteogenic and adipogenic differentiation of CYT, DAU and VIN treated MSC immediately after treatment. However the differentiation ability was regained in CYT and VIN treated MSC when the cells were cultured in drug free media. Reduced adipogenic differentiation of MSC was also reported after bleomycin treatment [20]. Several studies observed a toxic effect of chemotherapy on bone marrow derived stromal cells where decreased number of osteoprogenitors was reported in patients who underwent high dose chemotherapy with 5-fluorouracil, epidoxorubicin, and cyclophosphamide [2]. Bone marrow damage was seen in patients treated with 6-mercaptopurine and methotrexate [6] but MSC differentiation ability was unaffected during cisplatin treatment [19]. However, MSC treated with dexamethasone and vincristine recovered their proliferation and differentiation ability faster than other chemotherapeutic agents such as CYT [14] as seen also in our study. We found a significant downregulation of several cell surface receptors including CD44 after DAU treatment which the cells eventually recovered after the removal of the drug. Similar downregulation of CD44 expression in MSC was reported after cyclophosphamide and melphalan treatment [10].

An important aspect of our study was to understand how the MSC exposed to chemotherapeutic drugs protected the leukemia cells during treatment with the chemotherapeutic drugs. The chemoprotective effect of stromal cells in leukemia were reported by us and others [8, 13, 16, 30]. In the current study, we found a significant reduction in drug induced apoptosis of leukemia cells when they were co-cultured with MSC pre-exposed to the drug compared to the control MSC, suggesting an enhanced protective mechanism exhibited by these cells. Similar effect was observed in cisplatin pre-treated MSC, where they chemoprotected the breast cancer cells during cisplatin treatment [26]. We observed that drug treated MSC had increased expression of IL6, IL8 and FGF2 which was also seen in the cisplatin treated MSC [26]. A recent study suggested that increased levels of IL6, TNFA, bFGF, etc. in MSC might be involved in leukemia cells proliferation and chemoprotection [5]. We found in our study that, an increased level of FGF2 was sufficient to chemoprotect leukemic cells from DAU induced cell death and similar results were reported by Traer et al. where they found FGF2 mediated imatinib resistance in CML cells [29].

## Conclusions

Chemotherapeutic drug treatment affected the phenotype, osteogenic and adipogenic differentiation potential of MSC, and it modified the gene expression profile of the stromal cells to render them more chemoprotective of the leukemia cells. Thus, additional therapeutic efforts to target the stromal cell population will help in preventing chemoresistance, disease relapse in leukemia and to maintain a healthy bone marrow stroma.

## Additional files

Additional file 1: Table S1. Dose response curve and chemoprotection of leukemia cells by MSC. **a-c** Dose response curve showing percentage of live cells after treatment with indicated concentrations of CYT, DAU and VIN after 48 h. **d** THP1 leukemia cells were cultured for 48 h in the absence of MSC (CON) or in the presence of MSC (+MSC) or in the presence of drug pre-treated MSC (+PRE-TR MSC). The cells were treated with CYT (10mM), DAU (0.1mM) for 48 h and apoptosis percentage was analyzed flow cytometrically. Values are mean+SD, *n*=3 samples. *p < 0.05, **p < 0.005. (DOCX 12 kb)

Additional file 2: Figure S1. Dose response curve and chemoprotection of leukemia cells by MSC. **a-c** Dose response curve showing percentage of live cells after treatment with indicated concentrations of CYT, DAU and VIN after 48 h. **d** THP1 leukemia cells were cultured for 48 h in the absence of MSC (CON) or in the presence of MSC (+MSC) or in the presence of drug pre-treated MSC (+PRE-TR MSC). The cells were treated with CYT (10mM), DAU (0.1mM) for 48 h and apoptosis percentage was analyzed flow cytometrically. Values are mean+SD, n=3 samples. *p < 0.05, **p < 0.005. (DOCX 68 kb)

## Abbreviations

ACTC1: Alpha cardiac actin
BAX: Bcl-2-associated X protein
BCL2: B-cell lymphoma 2
CAT: Catalase
CYT: Cytarabine
DAU: Daunorubicin
FGF2: Fibroblast growth factor 2
GAPDH: Glyceraldehyde 3-phosphate dehydrogenase
IL6: Interleukin 6
IL8: Interleukin 8
MFI: Mean fluorescence intensity
MNSOD: Manganese superoxide dismutase
MSC: mesenchymal stem cells
OCN: Osteocalcin
PPARG: Peroxisome proliferator activated receptor gamma
PI: Propidium iodide
RBC: Red blood cells
SOX9: SRY (sex determining region Y)-box 9
TNFA: Tumor necrosis factor alpha
VIN: Vincristine
XIAP: X-linked inhibitor of apoptosis protein
