# Supplementary material for: Mesenchymal stem cells show functional defect and decreased anti-cancer effect after exposure to chemotherapeutic drugs
Source: J Biomed Sci. 2018 Jan 19;25:5. doi: 10.1186/s12929-018-0407-7 (PMC5774172; doi:10.1186/s12929-018-0407-7)
Supplement: Supplementary file 1 — Dose response curve and chemoprotection of leukemia cells by MSC. a-c Dose response curve showing percentage of live cells after treatment with indicated concentrations of CYT, DAU and VIN after 48 h. d THP1 leukemia cells were cultured for 48 h in the absence of MSC (CON) or in the presence of MSC (+MSC) or in the presence of drug pre-treated MSC (+PRE-TR MSC). The cells were treated with CYT (10mM), DAU (0.1mM) for 48 h and apoptosis percentage was analyzed flow cytometrically. Values are mean+SD, n=3 samples. *p < 0.05, **p < 0.005. (DOCX 12 kb) [file 12929_2018_407_MOESM1_ESM.docx]

**Supplementary table 1: List of primers**

| **Gene** | **Forward primer** | **Reverse primer** |
| --- | --- | --- |
| ACTC1 | 5′-GACGAGGAGACCACCGCCCT-3′ | 5′-TCCTCGGGAGCCACACGGAG-3′ |
| BAX | 5′-AACTGGACAGTAACATGGAG-3′ | 5′-TTGCTGGCAAAGTAGAAAAG-3′ |
| BCL2 | 5′-AACATCACAGAGGAAGTAGAC-3′ | 5′-CACTTGATTCTGGTGTTTCC-3′ |
| CAT | 5′-GCCTGGGACCCAATTATCTT-3′ | 5′-GAATCTCCGCACTTCTCCAG-3′ |
| FGF2 | 5′-AAGAGCGACCCTCACATCAA-3′ | 5′-CAGTTCGTTTCAGTGCCACA-3′ |
| IL6 | 5′-CTGACCCAACCACAAATGCC-3′ | 5′-GTTGTCATGTCCTGCAGCCA-3′ |
| ILF2 | 5′-CTTCCTTCAGTGAGGCCTTG-3′ | 5′-TAGGATCCCACCTGTCGAAC-3′ |
| MNSOD | 5′-CTGGACAAACCTCAGCCCTA-3′ | 5′-CTGATTTGGACAAGCAGCAA-3′ |
| SOX9 | 5′-AGTACCCGCACTTGCACAAC-3′ | 5′-CGTTCTTCACCGACTTCCTC-3′ |
| TNFA | 5′-CAGCCTCTTCTCCTTCCTGAT-3′ | 5′-ATGAGGTACAGGCCCTCTGAT-3′ |
| XIAP | 5′-ATTCACTTGAGGAGTGTCTG-3′ | 5′-CAAGTGATTTATAGTTGCTCCC-3′ |
