# Supplementary material for: Mesenchymal stem cells show functional defect and decreased anti-cancer effect after exposure to chemotherapeutic drugs
Source: J Biomed Sci. 2018 Jan 19;25:5. doi: 10.1186/s12929-018-0407-7 (PMC5774172; doi:10.1186/s12929-018-0407-7)
Supplement: Supplementary file 2 — Dose response curve and chemoprotection of leukemia cells by MSC. a-c Dose response curve showing percentage of live cells after treatment with indicated concentrations of CYT, DAU and VIN after 48 h. d THP1 leukemia cells were cultured for 48 h in the absence of MSC (CON) or in the presence of MSC (+MSC) or in the presence of drug pre-treated MSC (+PRE-TR MSC). The cells were treated with CYT (10mM), DAU (0.1mM) for 48 h and apoptosis percentage was analyzed flow cytometrically. Values are mean+SD, n=3 samples. *p < 0.05, **p < 0.005. (DOCX 68 kb) [file 12929_2018_407_MOESM2_ESM.docx]

**Additional file 2.**


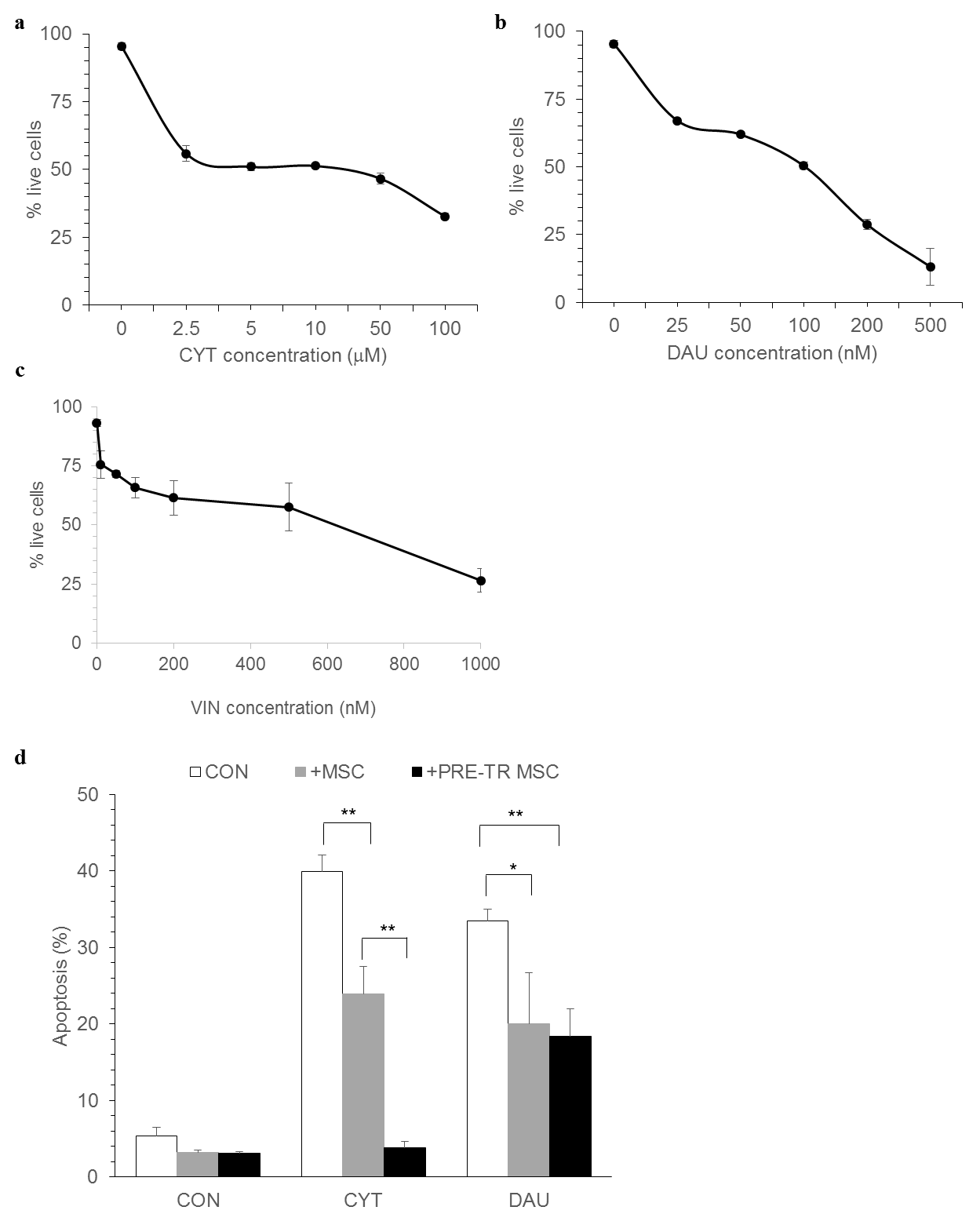


**Figure S1.** Dose response curve and chemoprotection of leukemia cells by MSC. **a-c**  Dose response curve showing percentage of live cells after treatment with indicated concentrations of CYT, DAU and VIN after 48 hours. **d** THP1 leukemia cells were cultured for 48 hours in the absence of MSC (CON) or in the presence of MSC (+MSC) or in the presence of drug pre-treated MSC (+PRE-TR MSC). The cells were treated with CYT (10μM), DAU (0.1μM) for 48 hours and apoptosis percentage was analyzed flow cytometrically. Values are mean+SD, n=3 samples. *p<0.05, **p<0.005.
